# Microbial contamination of traditional liquid herbal medicinal products marketed in Mwanza city: magnitude and risk factors

**DOI:** 10.11604/pamj.2016.23.65.7917

**Published:** 2016-03-01

**Authors:** Clementine Walther, Karol Julius Marwa, Jeremiah Seni, Peter Hamis, Vitus Silago, Stephen Eliatosha Mshana, Mary Jande

**Affiliations:** 1 School of Pharmacy, Catholic University of Health and Allied Sciences, Mwanza, Tanzania; 2 Weill Bugando School of Medicine, Catholic University of Health and Allied Sciences, Mwanza, Tanzania

**Keywords:** Microbial contamination, traditional liquid herbal medicinal products, Mwanza

## Abstract

**Introduction:**

The use of the traditional herbal medicinal products (THMPs) has been increasing worldwide due to the readily availability of raw materials and low cost compared to the synthetic industrial preparations. With this trend in mind, the safety and quality of THMPs need to be addressed so as to protect the community. The present study evaluated the magnitude and risk factors associated with microbial contamination of liquid THMPs marketed in Mwanza.

**Methods:**

A cross-sectional study was conducted in Mwanza city involving 59 participants from whom 109 liquid THMPs were collected and processed following the standard operating procedures. The data were analyzed using STATA software version 11.

**Results:**

The median age (interquartile range) of participants was 35 (27-43) years, with males accounting for 36 (61%). Of 109 liquid THMPs collected, 89 (81.7%) were found to be contaminated; with predominant fecal coliforms being *Klebsiella spp* and *Enterobacter* spp. fortunately, no pathogenic bacteria like *Salmonella spp* and *Shigella spp* were isolated. There was a significant association of liquid THMPs contamination with low education level (p< 0.001), lack of formal training on THMPs (p = 0.023), lack of registration with the Ministry of Health (p = 0.001), lack of packaging of products (p < 0.001) and use of unboiled solvents during preparation of THMPs (p < 0.001).

**Conclusion:**

There is high contamination rate of liquid THMPs in Mwanza City which is attributable to individuals and system-centered factors. Urgent measures to provide education to individuals involved in THMPs as well as setting up policies and regulations to reinforce THMPs safety is needed.

## Introduction

Contamination is the undesired introduction of impurities (chemical or microbiological or foreign matter) onto a starting material, intermediate product or finished herbal product during production, packaging, storage or transport of this product [1]. Generally, the presence of coliforms in traditional herbal medicinal product (THMPs) implies the possibility of recent fecal contaminations and inadequate sanitation measures in the cascade of THMPs production process; in this regard bacteria in the family enterobacteriaceae are predominantly implicated [1–4]. Moreover, some microbial contaminants can change the physicochemical characteristics which can then lead to harmful changes to the quality of THMPs [1]. The use of THMP is increasing worldwide due to the readily availability of raw materials and low cost compared to the synthetic industrial preparations [5–7]. This rapid expanding markets of THMPs use is apparently calling for scrutinizing issues related to safety and quality of these products [1, 5]. It is well know that THMPs microbial contamination can be a potential source of infections which in turn can result into a wide range of complications from gastroenteritis, sepsis, blindness and even death [5, 8, 9]. So, to prevent these undue complications, the World Health Organization (WHO) requires all local and international regulatory bodies to set up policies to ensure that THMPs meet the recommended standard of safety prior to be legalized for human consumption but this has remained an ideal situation as most of countries are still far from achieving this goal [1, 7]. There are limited studies addressing traditional herbal medicines′ contamination in Tanzania to guide regional specific interventions and a few of these studies were based in Dar es salaam only [4, 10]. Therefore, the present study evaluated the microbial contamination of liquid THMPs marketed in Mwanza city so as to inform relevant stakeholders for appropriate safety interventions.

## Methods

### Study site, design and sampling process

This cross sectional study was conducted in Mwanza city from June to July 2015 involving different manufacturers and local venders of liquid THMPs. A total of 59 voluntarily consented participants were enrolled serially and from these 109 non-repetitive liquid THMPs were collected.

### Data collection and laboratory procedures

Manufacturers and/or local venders for liquid THMPs were informed in details the aim of the study and then requested to voluntarily participate. The socio-demographic characteristics were collected using structured questionnaire. Then, liquid THMPs were collected in their original packages and taken to the Catholic University of Health and Allied Sciences (CUHAS) multipurpose laboratory for analysis. Using a spread plate technique, 0.1ml of liquid THMP was inoculated using the standard calibrated wire loop into Blood agar (HI MEDIA, Mumbai, India) and MacConkey agar (HI MEDIA, Mumbai, India). The culture plates were incubated at 35-370C for 24 hours. Bacterial isolates were quantified based on the standard laboratory procedures by counting the absolute number of similar colonies and then computing the respective equivalence of colony forming unit per milliliter of liquid THMPs (CFU/ml). The cut off point for contamination was taken as ≥ 10^2^ CFU/ml [3, 10]. The biochemical tests that were used to identify different bacterial species were lactose fermentation on MacConkey agar, triple sugar iron (TSI), sulphur indole motility (SIM), citrate, urease and oxidase tests [11].

### Data analysis

Data was entered into Excel sheet for consistence check and then exported to the STATA software version 11 for analysis according to the objectives of the study. Continuous variables were described as median (interquartile range). Categorical variables were described as proportion and were analyzed to compare the significance of difference in distribution THMPs contamination with variables using Chi-square test or Fischer's exact test where appropriate. The difference in distribution was considered significant if p-value was less than 0.05.

### Ethical considerations

This research sought ethical clearance from CUHAS/BMC institutional review board (No. 035/2015). The permission to conduct the research was asked from relevant Mwanza City authorities. Informed written consent was requested from each study participant prior to obtain information and liquid THMPs. Study participant were given code to ensure anonymity.

## Results

### Socio-demographic information of the study participants

Of 59 participants recruited in this study, males accounted for 36 (61%). The median age was 35 (27-43) years with the youngest participant aged 16 years and the oldest was 51 years old. Majority of participants were from Ilemela district 29 (49.2%) and 21 (35.6%) of the participants were secondary school leavers. Among all participants 11 (18.6%) reported to have formal training on THMPs and of these only 5 (8.5%) were registered by the Ministry of Health (Table 1).

**Table 1:** Socio-demographic information of the study participants

Socio-demographics		Frequency	Percentage (%)
**Sex**	Female	23	39
	Male	36	61
**Residence**	Ilemela	29	49.1
	Nyamagana	28	47.5
	Others*	2	3.4
**Education**	Illiterate	14	23.7
	Primary	18	30.5
	Secondary	21	35.6
	VETA**	6	10.2

* Usagara (1); Kisesa (1).

** Vocational education and training authority

### Baseline description of liquid THMP marketed in Mwanza city

Most of the participants were the manufacturers and buyers, 28 (47.5%) and were selling both liquid and powder forms of the THMPs, 56 (94.9%). More than three quarter of participants did not have special containers for storage of liquid THMPs. Moreover, most of the participants used unboiled tap water as solvent, 37 (63.4%) (Table 2).

**Table 2:** Description of liquid THMPs marketed in Mwanza city

Variable		Frequency	Percentage
**Status of participant**	Manufacturer & Buyer	28	47.5
	Buyer only	9	15.3
	Manufacturer only	22	37.3
**Formulation**	Liquid	3	5.1
	Liquid & Powder	56	94.9
**Packaging**	No special container	46	77.9
	Special container	13	22
**Solvent Source**	Mineral water	9	15.5
	Unboiled tap water	37	63.8
	Unboiled well water	6	10.3
	Others^¥^	6	10.3

¥ Sheep oil (2); Anywhere (2); Field water (1); Unspecified source (1)

### Proportion of liquid THMPs contaminated by fecal coliforms

**Figure 1:**
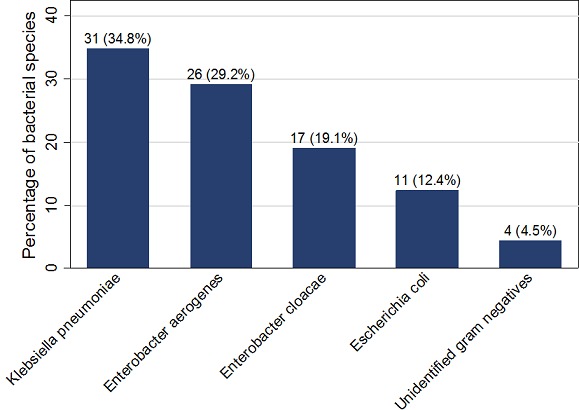
Bacterial species contaminating liquid THMPs in Mwanza city

A total of 109 liquid THMPs were collected from 59 different participants. Of these, 89 (81.7%) were found to be contaminated with fecal coliforms from approximately 72.9% (43/59) participants. Majority of the liquid THMPs had more than > 10^4^ CFU/ml (87.6%) whereas only 9.0% and 3.4% had 10^3^ CFU/ml and 10^2^ CFU/ml respectively. The most common bacterial species contaminating liquid THMPs were *Klebsiella pneumonia* and *Enterobacter aerogenes* in approximately 31 (34.8%) and 26 (29.2%) respectively. Fortunately, no pathogenic bacteria like *Salmonella spp* and *Shigella spp* were isolated in the present study (Figure 1).

### Risk factors associated with liquid THMPs contamination

There was a significant association between education level and the contamination of THMPs as 100% and 88.8% of the liquid THMPs from illiterate and primary school leavers were also contaminated by different bacterial species (p < 0.001). The level of contamination of liquid THMPs was also associated with participants with no formal training on THMPs, participants not registered by the Ministry of Health and Social Welfare (MoHSW), participants whose products were not packed as well as participants who used unboiled solvents (p-values of 0.023, 0.001, 0.001 and 0.001 respectively (Table 3).

**Table 3:** Association of liquid THMPs contamination with variables among study participants

Variable	Liquid THMPs contamination among participants (N = 59)		p-value
	No = 16 n (%)	Yes = 43 n (%)	
Sex	F	6 (26.1)	17 (73.9)	0.887
	M	10 (27.8)	26 (72.2)	
Education	Illiterate	0 (0.0)	14 (100)	<0.001
	Primary	2 (11.1)	16 (88.8)	
	Secondary	8 (38.1)	13 (61.9)	
	VETA^*^	6 (100)	0 (0.0)	
Residence	Ilemela	12 (41.4)	17 (58.6)	0.060
	Nyamagana	4 (14.3)	24 (85.7)	
	Others^**^	0 (0.0)	2 (100)	
Training on THMPs	Yes	6 (54.6)	5 (45.4)	0.023
	No	10 (20.8)	38 (79.2)	
Registration with MoHSW ^***^	No	11 (20.4)	43 (79.6)	0.001
	Yes	5 (100)	0 (0.0)	
Status of participant	Manufacturer & buyer	6 (21.4)	22 (78.6)	0.154
	Manufacturer only	5 (22.7)	17 (77.3)	
	Buyer only	5 (55.6)	4 (44.4)	
Formulation	Liquid	0 (0.0)	3 (100.0)	0.380
	Liquid &powder	16 (27.1)	40 (72.9)	
Packaging	Special container	13 (100.0)	0 (0.0)	<0.001
	No special container	3 (6.5)	43 (93.5)	
Solvent	Unboiled tap water	8 (21.6)	29 (78.4)	<0.001
	Unboiled well water	0 (0.0)	6 (100.0)	
	Mineral water	8 (88.9)	1 (11.1)	
	Others^¥^	0 (0.00)	6 (100.0)	

* Vocational education and training authority

** Usagara (1); Kisesa (1)

*** Ministry of Health and Social Welfare

¥ Sheep oil (2); Anywhere (2); Field water (1); Unspecified source (1)

## Discussion

The present study has revealed that most of the liquid THMPs marketed in Mwanza city are contaminated by bacterial coliforms. The high contamination rates of traditional medicinal products were also reported from Kaduna-Nigeria, Nairobi - Kenya and Dhaka - Bangladesh [2, 3, 12]. The high rates of contamination may be due to lack of stringent regulation of THMPs in these developing countries as shown in the present study where majority of participants whose products were contaminated were not formally registered by the MoHSW. The unacceptably high contamination of liquid THMPs were shown to be due low education level, lack of formal training, poor packaging as well as unboiling solvent used to mix liquid THMPs. Unhygienic practices in the processing of THMPs, which can potentially contaminate these products were also reported in studies done in Dar es salaam, Tanzania and South Africa [4, 13]. As a matter of fact there is an urgent need to have specific policies and regulations addressing liquid THMPs safety which are specifically focused on prevention of microbial coliforms contamination through direct involvement manufacturers or venders of liquid THMPs in Mwanza city. The liquid THMPs in the present study were predominantly contaminated by members in the family enterobacteriaceae, the findings which are also similar to other previous studies in Kenya and Nigeria [2, 12, 14]. But contrary to these studies in Kenya and Nigeria where pathogenic bacteria such as *Salmonella spp* and *Shigella spp* were also isolated; it was fortunate that in the present study these pathogenic bacteria were not isolated. Moreover, the indicator species connoting recent fecal contamination (*Escherichia coli*) accounted for 12.4%% of all bacterial coliforms isolated. This is contrary to 65.3% reported in Nigeria [12]. The variation in the bacterial isolates may be related to the sources of the raw materials and solvents used to prepare liquid THMPs as well as the extent of environmental level of contamination. Nevertheless, the presence of the bacteria coliforms isolated in this study which are known to be normal flora of human gastrointestinal tract need to be further evaluated in terms of the antimicrobial susceptibility profiles and as to whether there is similarities between these isolates and multidrug resistant bacteria currently associated with severe forms of infections at Bugando Medical Center, Sekou Toure Regional Hospital and other health facilities in Mwanza City [15–18]. Moreover, based on the fact that *Klebsiella spp* and *Enterobacter spp* are among predominant pathogens in this city and have recently being associated with an outbreak at BMC; the need to further delineate the genotypic relatedness is emphasized in the context of infections control and prevention in this setting [15, 19].

### Limitations of the study

The study was focused on liquid THMPs marketed in Mwanza city, thus other non-liquid formulations marketed in Mwanza city which may also be potentially contaminated with bacteria coliforms were not evaluated.

## Conclusion

There is high level of bacterial coliforms contamination in liquid THMPs (81.7%) in Mwanza city with majority of them being due to *Klebsiella spp* and *Enterobacter spp*. The contamination of liquid THMPs were attributable to both individuals and system-centered risk factors. Therefore, there is an urgent need to have specific educative programs, policies and regulations addressing liquid THMPs safety which are specifically focused on prevention of microbial coliforms contamination, so as to prevent the possibility of these pathogens to be involved in deadly invasive infections. A large study with large sample size will be important to further show the extent of microbial contamination in other THMPs formulation as well as assessing the similarities between these pathogens and those implicated in causing infections.

### What is known about this topic

- THMPs’ use is increasing worldwide due to its readily availability and low cost compared to industrial synthetic products.
- There are limited studies addressing THMPs microbial contamination; and these were largely based in Dar es Salaam city in Tanzania.

### What this study adds

- The present study had delineated unacceptably high microbial contamination rate of THMPs in Mwanza city as well as risk factors associated with this contamination.
- This regional-specific information will be used by relevant stakeholders in setting up policies and regulations to reinforce THMPs safety by addressing both individual and system-centered risk factors identified in this study.
